# A Review of Computational Modeling in Wastewater Treatment Processes

**DOI:** 10.1021/acsestwater.3c00117

**Published:** 2023-08-24

**Authors:** M. Salomé Duarte, Gilberto Martins, Pedro Oliveira, Bruno Fernandes, Eugénio C. Ferreira, M. Madalena Alves, Frederico Lopes, M. Alcina Pereira, Paulo Novais

**Affiliations:** † CEB − Centre of Biological Engineering, University of Minho, Campus de Gualtar, 4710-057 Braga, Portugal; ‡ LABBELS − Associate Laboratory, 4710-057 Braga, Guimarães, Portugal; § ALGORITMI Centre, Department of Informatics, University of Minho, Campus de Gualtar, 4710-057 Braga, Portugal; ∥ Águas do Norte, Rua Dr. Roberto de Carvalho, no. 78-90, 4810-284 Guimarães, Portugal

## Abstract

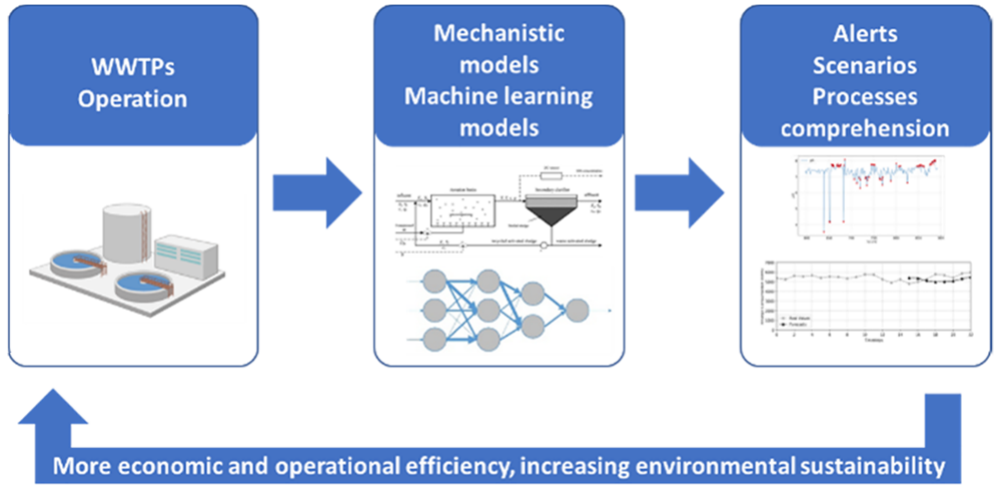

Wastewater treatment companies are facing several challenges related to the optimization of energy efficiency, meeting more restricted water quality standards, and resource recovery potential. Over the past decades, computational models have gained recognition as effective tools for addressing some of these challenges, contributing to the economic and operational efficiencies of wastewater treatment plants (WWTPs). To predict the performance of WWTPs, numerous deterministic, stochastic, and time series-based models have been developed. Mechanistic models, incorporating physical and empirical knowledge, are dominant as predictive models. However, these models represent a simplification of reality, resulting in model structure uncertainty and a constant need for calibration. With the increasing amount of available data, data-driven models are becoming more attractive. The implementation of predictive models can revolutionize the way companies manage WWTPs by permitting the development of digital twins for process simulation in (near) real-time. In data-driven models, the structure is not explicitly specified but is instead determined by searching for relationships in the available data. Thus, the main objective of the present review is to discuss the implementation of machine learning models for the prediction of WWTP effluent characteristics and wastewater inflows as well as anomaly detection studies and energy consumption optimization in WWTPs. Furthermore, an overview considering the merging of both mechanistic and machine learning models resulting in hybrid models is presented as a promising approach. A critical assessment of the main gaps and future directions on the implementation of mathematical modeling in wastewater treatment processes is also presented, focusing on topics such as the explainability of data-driven models and the use of Transfer Learning processes.

## 1 Introduction

Population growth and the change in the lifestyles and in the consumption patterns of humanity make it expectable that demand for water, energy, and other goods and services that require water will also increase, making this natural resource of primary importance with potential scarcity in some regions.^1^ Trying to overcome this issue, Sustainable Development Goal 6 of Agenda 2030 (of the United Nations) aims to ensure availability and sustainable management of water and sanitation for all, by 2030. Specifically, target 6.3 intends: “By 2030, improve water quality by reducing pollution, eliminating dumping and minimizing release of hazardous chemicals and materials, halving the proportion of untreated wastewater and substantially increasing recycling and safe reuse globally”.^2^ Therefore, concerns about the quality and quantity of clean water have been increasing. The improvement of the management of this natural resource has become one of the main research subjects nowadays.

A large part of the population live in urban centers where municipal authorities provide services and infrastructures to guarantee access to clean water to the population, through the urban water cycle, a challenge that includes disposal and treatment of effluents and water supply.^3,4^ To guarantee the water quality level, it is necessary to monitor its treatment in several wastewater treatment plants (WWTPs). Monitoring leads to the detection of failures in WWTPs, resulting in an improvement both in terms of quality and in reducing maintenance risks.^5−7^ Managing WWTPs is an exhaustive and complex process, as it depends on uncontrollable factors such as weather conditions or illicit discharges and water leaks. These factors cause variations in the flow and characteristics of the influent, requiring a more resilient and robust treatment. WWTPs aim to control all processes that ensure the quality of the water treatment, by minimizing simultaneously the environmental impacts and the operating costs.

Over the last few decades, computational models have gained recognition as effective tools for addressing some of these challenges by contributing to the economic and operational efficiencies of WWTPs. In order to predict the performance of WWTPs, numerous deterministic, stochastic, and time series-based models have been developed.^8,9^ These models can be used to predict the effluent parameters over the process and take preventive actions to avoid compromising its treatment quality.^10^ Predictive models are conceived to help decision-makers understand the data and make predictions about it to reduce environmental risks. Some examples of predictive models are artificial neural networks (ANNs), support vector machines (SVMs), and recurrent neural networks (RNNs), among others.

Besides the implementation of predictive models, modulation and detection of abnormal situations may also play important roles in WWTPs management. Anomaly detection for the cyber-physical system (CPS) is related to the identification of unfamiliar patterns of behaviors, i.e., the detection of potential intrusions as a deviation from normality (anomaly detection) that are not exhibited under normal operation.^6,11^ These anomalies could result from the physical environment and human error, but also from standard bugs or incorrect or suboptimal configurations in the software.^11^ The detection of anomalies plays a defensive role, at the same time that facilitates development, maintenance, and repairs of CPSs.^11^ Deep neural networks (DNNs) and SVMs are some examples of models that can be used for anomaly detection.

Mechanistic models, incorporating physical and empirical knowledge, are dominant as predictive models. Nevertheless, this type of model represent a simplification of reality, which results in an uncertainty of the models’ structure.^12^ With a constant increase in the amount of available data, data-driven methods are becoming more and more attractive. In this kind of model, the structure is not explicitly specified, but it is instead determined by searching for relationships in the available data.^12^

Over the last several years, some reviews on the application of AI models to water/wastewater treatment have become available, providing a systematic overview of the application of AI mainly in technology, both physical/chemical^13^ and biological^14,15^ treatments, and management.^16^ For example, Safeer et al.^13^ reviewed the recent advancements and applications of AI in water purification and wastewater treatment processes. Regarding water purification, this review emphasizes specific processes such as coagulation/flocculation, disinfection, membrane filtration, and desalination. Regarding the AI models for wastewater treatment, it focuses on membrane processes, and heavy metals and dyes.^13^ The paper by Sundui et al.^14^ explores the advancements and perspectives on utilizing ML algorithms to improve biological wastewater treatment processes, specifically in algae–bacteria consortia systems. The work of Singh et al.^15^ focuses on the application of AI and ML techniques for monitoring and designing biological wastewater treatment systems. In the case of Fu et al.^16^ their paper is a critical review of the role of deep learning in the field of urban water management. Since deep learning is a subset of ML, this is focused on only a part of the ML models. Nevertheless, it discusses broader aspects related to water management: water supply and distribution systems; urban flooding; cyber security; etc. Their review presents only a short section regarding wastewater treatment plants. Zhong et al.^17^ explores the innovative ideas and tools that have emerged with the adoption of ML techniques to address various environmental challenges in the field of environmental science and engineering, presenting a broader view of the application of ML when compared with our review. These authors approach only a subsection regarding the modeling of biochemical wastewater treatment systems.

Nevertheless, when comparing these reviews with the present work, we believe ours presents a wider perspective on wastewater treatment systems discussing both MM and ML to study factors such as effluent characteristics (Table 1), wastewater inflow rates (Table 2), anomaly detection (Table 3), and energy consumption optimization (Table 4) in WWTPs. This review also includes a section where the recent developments of hybrid models in wastewater treatment modeling are explored, since we believe that hybrid models that join the best of both (ML e MM) models are the best solution to improve model performance and model explainability. Finally, the main gaps and weaknesses, such as data size/periodicity, lack of transparency and explainability (blackbox approach), difficulty in predicting and responding to process disturbances, and the lower benchmark calculations until the moment are critically discussed.

**Table 1:** Summary of Parameters and Conditions of Studies Focused on Predicting WWTPs Effluent Operational Parameters

AI algorithm	input variable	output variable	objective	model performance	cross validation	overfitting control	correlation among input variables	ref
ANN	month, volumetric flow rate of inflow, pH, temp., CODd, TSS, TNinf, TN of pretreated food waste leachate	TNeff	Machine learning models to predict 1-day interval TNeff	0.55 (*R*^2^)/ 0.56 (NSE)	no	no	no	(74)
SVM				1.00 (*R*^2^)/ 1.00 (NSE)				
ANFIS	pHinf, CODinf, TSinf, NH_4_^+^ free, NH_4_^+^-N and TKNinf	TKNeff	SVM and ANFIS for predicting the TKN removal from a domestic WWTP	GBELL MF -TKNeff: 0.128 mg/L (RMSE)	yes	yes	yes; effluent TKN at the time (*t*) is strongly correlated with the TKNinf; NH_4_^+^-N and the NH_4_^+^ free	(8)
				Trapezoidal MF -TKNeff: 0.532 mg/L (RMSE)				
SVM				TKNeff: 0.155 mg/L (RMSE)				
FFNN	pHinf, conductivity (Condinf), BODinf, CODinf and TNinf	BODeff, CODeff, and TNeff	(i) AI based models and conventional multilinear models for prediction of the WWTP performance considering different combinations of input parameters	BODeff: 0.0065 (RMSE)	yes; employed the holdout (leave-group-out)	yes	no	(73)
				CODeff: 0.0014(RMSE)				
				TNeff: 0.0004 (RMSE)				
ANFIS				BODeff: 0.0053 (RMSE)				
			(ii) 3 ensemble techniques using the outputs of single models in order to improve the overall efficiency of the prediction performance	CODeff: 0.0012 (RMSE)				
				TNeff: 0.0005 (RMSE)				
SVM				BODeff: 0.0080 (RMSE)				
				CODeff: 0.0047 (RMSE)				
				TNeff: 0.0013 (RMSE)				
MLR				BODeff: 0.0077 (RMSE)				
				CODeff: 0.0014 (RMSE)				
				TNeff: 0.0006 (RMSE)				
CNN-LSTM	Temp(inf), pHinf, NH_3_inf, inflow, CODinf	Model 1: sewage inflow and the COD concentration (COD mass flow can be calculated from the prediction)	COD mass flow prediction model based on a deep learning algorithm	Model 1:48.0592 (RMSE)	no	yes, epoch adjustment	no	(78)
				Model 2:17.50 (RMSE)				
CNN		Model 2: predicts the COD mass flow directly		23.86 (RMSE)				
LSTM				29.88 (RMSE)				
FFNN	pHinf, TSSinf, BODinf, CODinf at the current time (t) and BODeff and CODeff at the previous time (t-1)	BODeff and CODeff at time *t*	AI models used for predicting BODeff and CODeff	BOD: 0.0341 (RMSE)	no	yes	yes	(77)
				COD: 0.0299 (RMSE)				
ANFIS				BOD: 0.0296 (RMSE)				
				COD: 0.0272 (RMSE))				
SVR				BOD: 0.0346 (RMSE)				
				COD: 0.0322 (RMSE)				
ARIMA				BOD: 0.0345 (RMSE)				
				COD: 0.0338 (RMSE)				
ANN	BOD5inf, DOinf, CODinf, Temp(inf), TSSinf, turbidity (inf), and Ecinf	BODeff, CODeff, and TSSeff	ANN and M5 model tree for assessing the performance of WWTP and estimating the quality of effluent	BODeff: 3.50 mg/L (RMSE)	no	no	yes	(75)
				CODeff: 3.43 mg/L (RMSE)				
				TSSeff: 2.62 mg/L (RMSE)				
M5 model tree				BODeff: 4.75 mg/L (RMSE)				
				CODeff: 4.74 mg/L (RMSE)				
				TSSeff: 4.60 mg/L (RMSE)				
SARIMAX	TPeff	TPeff	This study aims to explore the application of ML models on big data for prediction of wastewater quality from different full-scale WWTP	WWTP A: 0.01008 (MAE)	no	yes	yes	(79)
				WWTP B: 0.00530 (MAE)				
				WWTP C: 0.01104 (MAE)				
GTB				WWTP A: 0.01294 (MAE)				
				WWTP B: 0.00724 (MAE)				
				WWTP C: 0.01355 (MAE)				
RF				WWTP A: 0.01276 (MAE)				
				WWTP B: 0.00694 (MAE)				
				WWTP C: 0.01321 (MAE)				
SVM				WWTP A: 0.01290 (MAE)				
				WWTP B: 0.00694 (MAE)				
				WWTP C: 0.01233 (MAE)				
LSTM				WWTP A: 0.01176 (MAE)				
				WWTP B: 0.00645 (MAE)				
				WWTP C: 0.01337 (MAE)				
ANFIS				WWTP A: 0.01417 (MAE)				
				WWTP B: 0.00780 (MAE)				
				WWTP C: 0.01488 (MAE)				
FFNN	inflow, outflow, CODinf, NH3inf, TNinf, TPinf, pHinf, CODeff, NH3eff, TNeff and TPeff	CODeff and TNeff	The goal of this study is to predict in real time, the water quality of WWTP, by using an improved FFNN coupled with an optimization algorithm	CODeff: 6.3% (MAPE)	yes	yes	no	(76)
				TNeff: 3.6% (MAPE)				
IFFNN				CODeff: 5.9% (MAPE)				
				TNeff: 2.8% (MAPE)				
Ga-IFFNN				CODeff: 3.7% (MAPE)				
				TNeff: 0.6% (MAPE)				

**Table 2:** Summary of Parameters and Conditions of Studies Focused on Predicting WWTPs Inflow

AI algorithm	input variable	output variable	objective	model performance	cross validation	overfitting control	correlation among input variables	ref
MLP-ANN	influent data, rainfall data, and radar reflectivity data	Influent flow	Neural network approach is used to predict influent flow in the WWTP	t: 1.09 (MAE)	no	no	no	(87)
				t+15:1.48 (MAE)				
				t+30:1.89 (MAE)				
				t+60:2.75 (MAE)				
				t+90:3.61 (MAE)				
				t+120:4.46 (MAE)				
				t+150:5.26 (MAE)				
				t+180:6.02 (MAE)				
SVM	rainfall values, the water levels of the Wisłok river, and WWTP sewage entrances	inflow	Different approaches of data mining to model the inflow of sewage into the WWTP	Q(t-1): 2.963 (MAE)	no	no	yes	(93)
				P(t-1): 4.127 (MAE)				
				h(t-1): 3.467 (MAE)				
				Q(t-1), h(t-1): 2.854 (MAE)				
				P(t-1), P(t-2): 4.011 (MAE)				
				h(t-1), h(t-2): 3.551 (MAE)				
				Q(t-1), Q(t-2): 2.815 (MAE)				
				P(t-1), h(t-1): 2.966 (MAE)				
				Q(t-1), P(t-1) 2.912 (MAE)				
				Q(t-1), Q(t-2), h(t-1): 2.789 (MAE)				
				Q(t-1), Q(t-2), h(t-1), P(t-1): 2.647 (MAE)				
				Q(t-1), Q(t-2), h(t-1), P(t-1), P(t-2): 2.641 (MAE)				
RF				Q(t-1): 2.859 (MAE)				
				P(t-1): 4.127 (MAE)				
				h(t-1): 3.553 (MAE)				
				Q(t-1), Q(t-2): 2.767 (MAE)				
				P(t-1), P(t-2): 4.056 (MAE)				
				h(t-1), h(t-2): 3.507 (MAE)				
				Q(t-1), h(t-1): 2.847 (MAE)				
				P(t-1), h(t-1): 3.008 (MAE)				
				Q(t-1), P(t-1): 2.721 (MAE)				
				Q(t-1), Q(t-2), h(t-1): 2.786 (MAE)				
				Q(t-1), Q(t-2), h(t-1), P(t-1): 2.651 (MAE)				
				Q(t-1), Q(t-2), h(t-1), P(t-1), P(t-2): 2.617 (MAE)				
KNN				Q(t-1): 2.936 (MAE)				
				P(t-1): 4.509 (MAE)				
				h(t-1): 3.686 (MAE)				
				Q(t-1), Q(t-2): 2.965 (MAE)				
				P(t-1), P(t-2): 4.434 (MAE)				
				h(t-1), h(t-2): 3.696 (MAE)				
				Q(t-1), h(t-1): 2.961 (MAE)				
				P(t-1), h(t-1): 3.157 (MAE)				
				Q(t-1), P(t-1): 2.87 (MAE)				
				Q(t-1), Q(t-2), h(t-1): 2.995 (MAE)				
				Q(t-1), Q(t-2), h(t-1), P(t-1): 2.718 (MAE)				
				Q(t-1), Q(t-2), h(t-1), P(t-1), P(t-2): 2.682 (MAE)				
Kernel regression (K)				Q(t-1): 2.935 (MAE)				
				P(t-1): 4.189 (MAE)				
				h(t-1): 3.691 (MAE)				
				Q(t-1), Q(t-2): 2.842 (MAE)				
				P(t-1), P(t-2): 4.122 (MAE)				
				h(t-1), h(t-2): 3.939 (MAE)				
				Q(t-1), h(t-1): 3.152 (MAE)				
				P(t-1), h(t-1): 3.408 (MAE)				
				Q(t-1), P(t-1): 3.193 (MAE)				
				Q(t-1), Q(t-2), h(t-1): 3.100 (MAE)				
				Q(t-1), Q(t-2), h(t-1), P(t-1): 3.002 (MAE)				
				Q(t-1), Q(t-2), h(t-1), P(t-1), P(t-2): 2.925 (MAE)				
RF	historical weather data: max temp., min temp., mean temp., heating degree days, cooling degree days, total rain, total snow, total precipitation, and accumulated precipitation	daily wastewater inflow	RF for wastewater inflow prediction	confidential WWTP: 35.937 (RMSE)	yes	no	Weather parameters were selected for each WWTP according to a correlation analysis and to the literature	(92)
				Humber WWTP: 7.547 (RMSE)				
MLP				confidential WWTP: 95.699 (RMSE)				
				Humber WWTP: 19.269 (RMSE)				
SVM	inflow rate, COD, BOD_5_, NH_4_^+^, and TKN	current weather condition	Soft-sensor for predicting the current weather signal	accuracy mean of 3^3^ validation data sets	yes	In the strong filter row, the authors obtained excellent accuracy rates. This was caused by an overfit to the training data, as explored in validation phase	COD and BOD5 are extremely correlated; NH_4_^+^ and TKN are very correlated; therefore inflow, COD and NH_4_^+^ were the selected variables	(86)
				no filter: 0.45				
				smooth filter: 0.68				
				strong filter: 0.33				
Gaussian Naive Bayes				no filter: 0.41				
				smooth filter: 0.56				
				strong filter:0.39				
DT				no filter: 0.45				
				smooth filter: 0.75				
				strong filter: 0.33				
KNN (1)				no filter: 0.46				
				smooth filter: 0.85				
				strong filter: 0.35				
KNN (3)				no filter: 0.46				
				smooth filter: 0.82				
				strong filter: 0.35				
RF				no filter: 0.47				
				smooth filter: 0.84				
				strong filter: 0.33				
MLP-ANN	TN, NH_4_^+^, BOD, COD, mixed liquor suspended solids (MLSS), Mixed liquor volatile suspended solid (MLVSS), pH, DO	TNinf	Feature selection methods for enhancing the prediction performance of TN in the WWTPs	scenario I: 77 × 10^–3^ (RMSE)	no	no	TN had a good correlation with NH_4_-N, COD, and BOD, and a weak correlation with pH and DO	(88)
				scenario II: 79 × 10^–3^ (RMSE)				
				scenario III: 74 × 10^–3^ (RMSE)				
				scenario IV: 73 × 10^–3^ (RMSE)				
RF				scenario I: 96 × 10^–3^ (RMSE)				
				scenario II: 60 × 10^–3^ (RMSE)				
				scenario III: 55 × 10^–3^ (RMSE)				
				scenario IV: 55 × 10^–3^ (RMSE)				
GBM				scenario I: 78 × 10^–3^ (RMSE)				
				scenario II: 72 × 10^–3^ (RMSE)				
				scenario III: 68 × 10^–3^ (RMSE)				
				scenario IV: 68 × 10^–3^ (RMSE)				

**Table 3:** Summary of Parameters of Studies Focused on Anomaly and Fault Detection in WWTPs

AI algorithm	target	objective	model performance (*F*-scores)	ref
DNN	sensors	Application of unsupervised machine learning to anomaly detection for a CPS	0.80281	(11)
SVM			0.79628	
DBM	influent conditions	Application of unsupervised machine learning to anomaly detection for a CPS	0.98 (OCSVM)	(96)
RBM			0.99 (OCSVM)	
RNN			0.97 (OCSVM)	
RNN-RBM			0.99 (OCSVM)	
Stand alone			0.98 (OCSVM)	
LSTM	WWTP sensor data	Method based on DNN (specifically, long short-term memory) compared with statistical and traditional machine learning methods	0.9267	(97)
PCA-SVM			0.8667	

**Table 4:** Summary of Parameters and Conditions of Studies Focused on Predicting and Performing Energy Consumption Optimization in WWTPs

AI algorithm	input variable	output variable	objective	model performance	cross validation	overfitting control	correlation among input variables	ref
NN	CODeff, TPeff, TNeff, BOD5eff, tCODinf, TPinf, TNinf, BOD_5_inf, Inflow, the price of energy and the removal performance of COD, TN, and TP	energy cost	ML was used to generate high-performing energy cost models for WWTP	*R*^2^ > 0.86	no	no	no	(98)
RF				*R*^2^ > 0.95				
ANFIS	DO, oxidation reduction potential (ORP), temp., NH_4_^+^, and NO_3_^–^ in the oxidation tank, and the output TN	airflow rate (Ua) and the internal recycle Qr	Development of a model capable of estimating the process variables, providing the right amount of aeration to achieve an economical and efficient operation	NO_3_^–^: 0.12 mg/L (MAE)	no	no	no	(100)
				NH_4_^+^: 0.04 mg/L (MAE)				
				Ua: 22.43 N m^3^/h (MAE)				
PCA-CNN-LSTM	energy consumption, material consumption, and influent conditions	BOD_5_eff, CODeff, Sseff, pHeff, TPeff, TNeff, NH_3_eff, *E. coli*, Mud vol	energy and materials-saving management via deep learning for WWTPs	BOD_5_eff: 1.2984 (RMSE)	no	no	yes	(101)
				CODeff: 3.5454 (RMSE)				
				SSeff: 2.4698 (RMSE)				
				pHeff: 0.8889 (RMSE)				
				TPeff: 0.0829 (RMSE)				
				TNeff: 2.9816 (RMSE)				
				NH_3_eff: 0.6784 (RMSE)				
				*E.coli*: 2.1633 (RMSE)				
				Mud vol: 1.4207 (RMSE)				
DNN	Temp. influent, recirculated sludge flow, influent flow	energy consumption	ANN for creating an optimal model of energy consumption in a WWTP	90–92% (*R*^2^)	no	no	no	(102)
RF	design treatment capacity, annual average load rate, and removal ratios (BODiinf/BODeff, CODinf/CODeff, NH_3_ifnf/NH_3_eff)	energy consumption	Energy consumption model of WWTPs through machine learning using data from 2472 WWTPs in China, employing the RF approach	0.106 kWh/m^3^ (RMSE)	no	no	yes	(103)
LSTM	influent flow, COD, and TN removed	energy consumption	Developing, tuning, and evaluation of a set of candidate DL models with the goal of forecasting the energy consumption of a WWTP, using a recursive multistep approach	Model 1 (Multi-Variate-Scenario 3): 729.73 (RMSE)	yes	yes	yes; the influent flow had the highest correlation coefficient with the target parameter	(99)
				Model 2 (Uni-Variate-Scenario 1): 913.90 (RMSE)				
GRU				Model 1 (Uni-Variate-Scenario 1): 715.42 (RMSE)				
				Model 2 (Uni-Variate-Scenario 1): 869.85 (RMSE)				
CNN				Model 1 (Multi-Variate-Scenario 3): 690.00 (RMSE)				
				Model 2 (Uni-Variate-Scenario 1): 869.78 (RMSE)				

## 2 Mechanistic Wastewater Models: A Piece of History

Water quality modeling has evolved since the early years of the 20th century. The pioneering work of Streeter and Phelps (1925)^18^ launched the basis for the evolution and development of mathematical models applied to water quality problems. Later, with the emergence of computational capabilities, it allowed the development of more complex models.

Mechanistic models or deterministic models implement a set of differential equations reflecting the mass balance equations and other conserved quantities, for all involved compounds.^19^ Back in 1987, Henze et al. developed the Activated Sludge Model No. 1 (ASM1), the first WWTP model well accepted by research community and industry.^20^ ASM1 describes the removal of nitrogen and organic carbon compounds, with the simultaneous consumption of electron acceptors (nitrate and oxygen), in municipal activated sludge WWTPs. In ASM1, the biological reactions are defined according to the Monod kinetics, and the majority of the basic concepts were inspired from the activated sludge model developed by Dold et al.^21^ This integrated model combined the chemical oxygen demand (COD) conservation with stoichiometry and kinetics, by expressing transformation rates in the form of derivatives.^21^

Further developments led to the expansion of the ASM model to include biological phosphorus removal and chemical phosphorus removal via precipitation processes, ASM2^22^ and ASM2d^23^ models, as well as the ASM3,^24^ which were intended to amend the ASM1 model flaws and facilitate the calibration. ASM2^22^ and ASM2d^23^ models include the description of biological P processes and chemical P removal via precipitation, with simultaneous nitrification-denitrification processes. Later, a new version of the ASM model, ASM3,^24^ was developed, intending to amend the ASM1 model flaws that have emerged during its usage. ASM3 has almost the same objectives as ASM1, and supposedly is easier to calibrate. This new ASM version distinguishes the importance of storage polymers in the conversion of heterotrophic activated sludge, which is mainly achieved by converting the circular growth–decay–growth model, frequently known as death–regeneration concept, into a growth-endogenous respiration model.^19^

To integrate all of these tools and guarantee their evaluation and comparison, several benchmark tools have been developed by Working Groups of COST Action 682 and 624, and later by the IWA Task Group of Benchmarking of Control Strategies. This benchmark platform defines the WWTP arrangement, the simulation model, influent data sets, test procedures, and evaluation criteria.^25^ The Benchmark Simulation Model no. 1 - BSM1^25^ was the first layout to be developed and is comprised by a five-compartment activated sludge reactor divided in two anoxic tanks and three aerobic tanks. It combines nitrification with predenitrification, which is usually used for nitrogen removal in municipal WWTP. BSM2^26^ was developed to also integrate the sludge treatment. Finally, a Risk Module was proposed,^27^ considering the microbiology-related settling problems (filamentous bulking sludge, filamentous foaming, or deflocculation), which cause several operational problems in WWTPs.^28^ Additionally, BSM–UWS (urban wastewater system), established as an integrated model library aiming to simulate on a single platform the dynamics of flow rate and pollutant loads in all the subsystems of an urban wastewater system, and the BSM2G for predicting greenhouse gas emissions were also developed.^29^ Benchmark calculations using ASM models offer several advantages.^30^ ASM models are based on a scientific understanding of the biological and chemical processes, thus providing insights into the underlying mechanisms and dynamics of the treatment process.^19^ They are flexible and can be customized to represent specific treatment configurations, operational conditions, and influent characteristics. ASM models can simulate the behavior of wastewater treatment processes and predict their performance under different scenarios, as well as quantify key performance indicators (KPIs), such as effluent quality, sludge production, nutrient removal efficiency, and energy consumption.^31^ Optimization and troubleshooting efforts, and the estimation of resource requirements are other advantages of benchmark calculations using ASM models.^30^ Nevertheless, ASM models also have some drawbacks such as model complexity, accuracy, data requirements, high uncertainty due to many simplifications and assumptions, lack of adaptability, insufficient model validation, and computational requirements.^32−34^

One of the main drawbacks of mechanistic models is the need for model calibration. Model calibration is the adjustment of model parameters starting from a default parameter set, which is updated considering the fitting of experimental data with simulation results. This is a time-consuming step and hinders the broader application of these models.^32^ In the calibration, it can be used nondynamic data (i.e.: composite 24 h samples) or dynamic data (dynamic profiles of influent and effluent composition).^22^ The calibration can be carried out following a heuristic approach, considering the process understanding and the model structure or through a purely mathematical optimization process.^19^ The first approach is more sensitive but requires a considerable level of expert knowledge of the process. Usually, the calibration process based on engineering (heuristic) approaches could be combined with the mathematical approach, by applying a sensitivity analysis to model parameters.^35^

In addition, despite ASM models being widely accepted, some novel treatment processes, such as anaerobic ammonium oxidation processes^36^ and membrane treatment,^37^ are still lacking for standard modeling frameworks.^34^ Also, digital twins or virtual replicas of water and wastewater treatment infrastructures have been developed. Some examples include simulation platforms such as EPANET for drinking water distribution network, collection systems (info works, SWMM) water-related domain (DHI) and water resources recovery facilities (Biowin, Aquasim, GPS-X, Sumo, Simba, WEST).^38^ However, the limited prediction capabilities of mechanistic models hinder its application.

**Figure 1:**
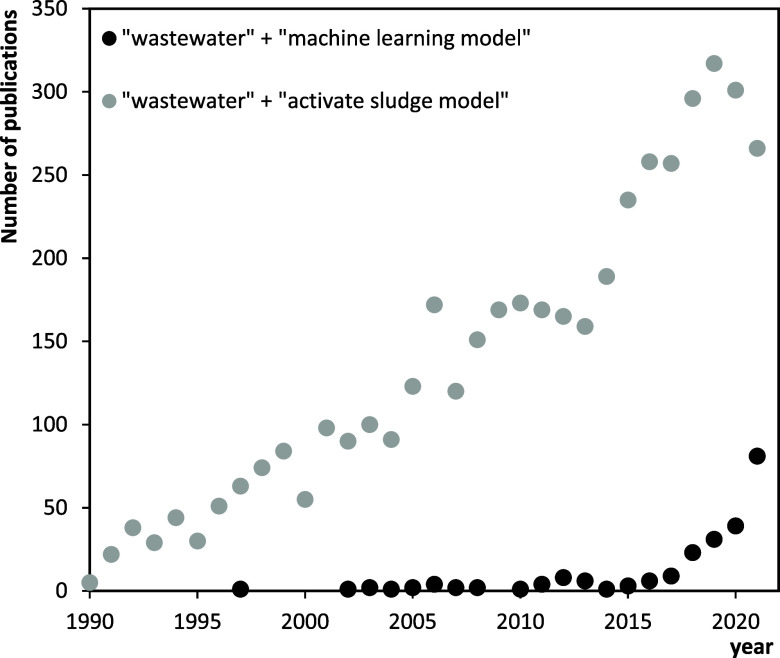
Evolution of the number of publications related to wastewater and mechanistic (activate sludge model) or machine learning model. Data for Figure 1 were obtained by analyzing the number of publications by year in the Web of Science database, using the following searching keywords “wastewater” + “machine learning model” and “wastewater” + activate sludge model”.

In summary, the long history of ASM models application has demonstrated their effectiveness for the design, optimization, and operation of WWTP, as well as in the comprehension of involved processes.^39^ In an attempt to adapt the models to changes in WWTPs, i.e., process upgrades and introduction of new treatments, or even more strict effluent discharge limits, new models and/or extensions to existing models have been developed.^33^ However, these changes result in an increase in the model complexity, making them too parametrized and difficult to calibrate.^40^ Therefore, their popularity has decreased over the last years, as can be observed in Figure 1, where the number of publications related to the Activate Sludge Model (one of the most used mechanistic models) is in decline. Although the mechanistic models represented by the ASM model have been widely used, in recent years, studying the wastewater treatment processes with the data-driven methods have gradually emerged and developed rapidly with the development of machine learning algorithms and the increase in the size of data sets (Figure 1). A recent review paper had also highlighted the explosive growth in the number of publications related with ML in the field of environmental science and engineering, being around 50% in water sector.^17^

## 3 Wastewater Treatment Modeling Using Machine Learning

ML has demonstrated in the last years to be a strong tool to simplify the modeling of WWTP processes.^10,41^ Through ML, machines can acquire knowledge to perform tasks usually associated with humans, considering what has been previously experienced. Thus, the development of models entirely identified based on input–output data without reflecting knowledge of physical, chemical, or biological processes in the model structure can be used to indicate the occurrence of possible problems in WWTPs, thus activating appropriate control actions when needed.

A significant difference between humans and computers is that humans can automatically change their behavior through learning from previous mistakes. Thus, the essence of Machine Learning (ML) is the creation of models and tools that can learn and consequently improve their performance, through continuous data collection, resulting in experience and expertise.^42,43^ In ML there are three distinct paradigms: supervised, unsupervised, and reinforcement learning. Supervised learning is an ML approach based on accumulated experiences incorporated into the training set. The system is programmed or trained from a predefined and well-classified set of data. After processing a series of information and learning from it, the program can decide when to receive new data records. The most used ML models are supervised,^44^ since this method may be used in situations where the analysis of historical data allows predicting possible future behaviors. Supervised learning has a wide range of application categories, such as the classification and regression method.^45^ Regarding classification, the program can make fewer complex decisions, such as indicating a positive or negative response. In the case of regression, the value to be predicted by the program follows a continuous spectrum and allows answering questions such as “how many are there” or “how much does it cost”.^46^ Decision trees (DTs), SVMs, and ANNs are some models present in this type of learning.^47−49^ ANNs, one of the models most used in the simulation and prediction of the performance of biological treatment in WWTP, use models composed of several artificial neurons, connected by links of variable weight, to form black box representations of pseudoneurological systems.^41^ Each neuron receives input signals from other neurons, processes them, and sends out the output, which in turn is passed on as input to subsequent neurons.^50^ The ANNs learn from training data and capture the relationships between data points, which can be used for simulation, prediction, and optimization. ANNs are a type of information processing system that resembles the human brain.^51^

On the other hand, in unsupervised learning, there is no feedback on the obtained results so that the model can use them as a reference for learning. In other words, there is no supervisor to tell us whether we are going the right way or not. Also, because the results are unknown, it becomes impossible to determine their accuracy, making supervised models more applicable to real-world problems. This technique is used based on observation and discovery. Such unsupervised learning is designed to be used in situations in which information about the desired results is unknown. The patterns discovered with unsupervised learning methods can be useful when implementing supervised machine learning methods. As an example, cluster analysis can be carried out by unsupervised techniques and use the cluster to which each row belongs as an additional resource in the supervised learning model.^52^ Some examples of unsupervised learning are the K-means and K-medoids models.^53^ Reinforcement learning allows computational agents to learn from interactions with the environment in which they are inserted. In the reinforcement learning paradigm, an agent is rewarded or punished, depending on the decision made. With the time and repetition of the teachings, the agent will learn the actions that generate a greater reward for each situation that the environment presents and thus avoid the actions that create punishments or smaller rewards. Contrary to what happens in most ML methods, the learner is not informed of the path he must take but rather determining which actions obtain the best reward by trying them. Moreover, actions can affect the immediate reward and subsequent ones.^54^ Q-Learning and SARSA are some examples of models used in reinforcement learning.^55^

With the increase in the amount of data available, deep learning (DL) emerged as a subarea of ML. With the emergence of more complex problems, the evolution in technology and hardware has enabled the use of DL models to solve these types of problems and improve existing solutions, such as image recognition or tomography analysis.^56^ The use of DL aims to imitate the functioning of the human brain in data processing, learn without human supervision, and use unstructured and unlabeled data, following possible approaches, supervised and unsupervised. Autoencoders and generative modeling are examples of the unsupervised approach,^57^ while MultiLayer Perceptrons (MLP), RNNs or convolutional neural networks (CNNs) are examples of the supervised approach.^56,58,59^ Considering the autoencoders, they reduce the dimensionality of the input through an encoder, reconstructing it again by a decoder. These models are evaluated by minimizing the reconstruction error.^60^ MLP networks are known as feedforward neural networks (FFNN), since each neuron in these networks can only be connected to units in the next layer and never in the previous layer. This makes the processing flow from input to output unidirectional, which differentiates it from other feedback networks, such as Hopfield networks. This type of network allows the creation of multiple hidden layers which allows the resolution of problems whose separation between classes is not linear.^61,62^ CNNs are specially developed for computer vision because the extraction of characteristics is done by the network itself, which is trained with it. This type of deep neuronal network is divided into two parts: the features extractor, which can be composed of convolution and reduction layers; and the classifier, composed of fully connected layers, as in an ANN. With CNN, the characteristics of input images are extracted through successive convolutions and resizing. These networks are easy to train and have fewer parameters than other fully connected networks.^63^ Despite this, in recent times, these networks have been used in the scope of time series forecasting, namely, through 1D-CNNs. The prediction of PM2.5 levels in the air and the river’s flow are some examples of the application of 1D-CNNs.^64,65^ RNNs constitute a class of networks in which the evolution of the state depends on the current input and the current state. This property makes it possible to perform context-dependent processing, allowing long-term dependencies to be learned. Signals supplied to a recurring network in an instant of time *t* can change the behavior of that network in the next moments (*t* + *k*, *k* > 0). These networks can have connections that return from the outgoing nodes to the incoming nodes, or even arbitrary connections between nodes.^66,67^ Special cases of RNNs are long short-term memory (LSTM) and gated recurrent units (GRUs).

The fuzzy logic (FL) algorithms are composed by the fuzzy inference system (FIS), fuzzification, defuzzification, and fuzzy rules, and were developed to model complex and imprecise systems.^68^ Of these compounds, the most used is the FIS, which is based on four functional blocks: the fuzzification unit, the decision-making unit, the knowledge base (which includes the rules and the database), and the defuzzification unit.^41^

Genetic algorithms (GAs) are evolutionary algorithms that use Darwin’s theory to model the natural evolutionary process to achieve the minimum or maximum objective function.^69−71^ Selection, crossover, and variation are the main principles of applying genetic operators to chromosomal populations.

Artificial neural networks - genetic algorithm (ANN-GA) use a GA to iteratively optimize the parameters in the neural network and increase its problem-solving power.

Neural-fuzzy (NF) systems use ANN learning algorithms to determine the parameters of FIS, sharing knowledge representations, and data structures. A common way to apply a learning algorithm to a fuzzy system is to represent it in a special ANN -like architecture.^72^

### 3.1 Practical Applications of Artificial Intelligence to Wastewater Treatment

The most common ML models used in the simulation, prediction, evaluation, and diagnose of wastewater treatment operations are the ANN, FL, GA, and NF, as well as ANN-GA as hybrid models.^10,41^

#### 3.1.1 Forecasting Effluent Parameters

Several AI models have been applied to predict WWTPs effluent characteristics. Table 1 summarizes the analyzed works. For example, the effluent biochemical oxygen demand (BOD), COD, and total nitrogen (TN) of Nicosia WWTP were predicted by FFNN, adaptive neuro fuzzy inference system (ANFIS), SVM, and a multilinear regression (MLR).^73^ ANFIS showed better results on calibration and verification phases in comparison to other models. Regarding BOD forecasting, the performance obtained by the AI model increased up to 14%, 20%, and 24%, taking into account the simple averaging ensemble (SAE), weighted averaging ensemble (WAE), and neural network ensemble (NNE), as ensemble models, respectively. For COD and TN, the performance efficiency increased only up to 5%.^73^ According to the authors, SVM was found to be more reliable than the MLR model, and single models should not be considered as a trustable model for the simulation of effluents BOD in WWTP. The models tested in this study responded satisfactorily and are recommended for the simulation of effluents’ COD and TN.^73^ Effluent TN from a WWTP in Ulsan, Korea, was also predicted by ANNs and SVMs models, with the SVM model showing a higher prediction accuracy during the training phase.^74^ However, the sensitivity analysis (Latin-Hypercube one-factor-at-a-time - LH-OAT) showed that the ANN model was a better model for 1-day intervals for the prediction of TN, regarding the cause effect relationship between TN concentration and modeling input values.^74^ Although ANN and M5 model tree revealed reliability, robustness, and high generalization capability, ANN (*R*^2^ equal to 0.95, 0.95, and 0.97 for BOD_5_, COD, and total suspended solids (TSS), respectively for model validation) showed better performance than M5 model tree (*R*^2^ of 0.88, 0.90, and 0.83 for BOD5, COD, and TSS, respectively for model validation) when applied to the WWTP of Ramin thermal power, Ahvaz Iran, covering 3 years (2013 to 2015) daily data set.^75^ The effluent total Kjeldahl nitrogen (TKN) concentration yielded from a WWTP was also predicted by SVM and ANFIS models, with SVM models providing more solid results than the ANFIS models. Among ANFIS models, the Gbell MF MODEL was found to be a little more efficient in modeling the nonlinear time series, being able to define the interrelation between various wastewater quality variables.^8^ Besides TN,^76^ ML was used to predict an effluent’s COD, from a WWTP in Jiangsu Province, China. The ML model was developed by joining an improved feed-forward neural network (IFFNN) with an optimization algorithm. The input variables for the model consisted of data of WWTP process monitoring and operation. When IFFNN was compared to traditional FFNN, the IFFNN enhanced prediction performance by 72.6% for TN and 52.3% for COD. The IFFNN model structure was optimized with a genetic algorithm (GA). The implementation of IFFNN helped to overcome the problem of overfitting when compared to the traditional FFNN. The GA-IFFNN model was able to predict TN with values very closed to the real data and was shown to be efficient in determining complex nonlinear relationships and extrapolation.^76^

Nourani et al.^77^ showed that the prediction accuracy of the black box AI model, composed by FFNN, support vector regression (SVR), and ANFIS, increased up to 20% at the verification phase, using jittering data preprocessing and postprocessing ensemble models to predict model parameters through an autoregressive integrated moving average (ARIMA) model. The model was used to predict the BOD and COD present in the effluent of Tabriz WWTP using the data from 2016 to 2018.^77^ These authors concluded that AI models are more suitable than ARIMA in the prediction of WWTP parameters. Hybrid models, such as CNN-LSTM model, presented better results than the CNN or LSTM stand-alone models, in the prediction of urban sewage COD, supporting the further development of feedforward control systems.^78^ A recent study used data of 10 parameters from 3 WWTPs that were collected hourly.^79^ In this work, the total phosphorus (TP) in the outlet was predicted by testing 6 ML models: seasonal autoregressive integrated moving average (SARIMAX), gradient tree boosting (GTB), random forest (RF), SVM, LSTM, and ANFIS. Despite having data from 10 parameters, the TP in the outlet (TPeff) was shown to be better than other variables to predict itself. SARIMAX showed the best prediction with acceptable computation efficiency, while LSTM presented a good performance but it was rather time-consuming.^79^ A big data set, with historical data from 2010 to 2020 of a WWTP, was used as input to a ML ensemble model that combines ANN, ANFIS, and SVR to predict 15 process parameters.^80^ According to the authors, the implementation of a multistage model structure resulted in the ability of predicting the intermediate parameters of the process which are affected by the influent characteristics, that can be useful to explain the overall process performance.^80^

Some of these predictive models were also implemented in processes for treating industrial wastewaters. For example, Picos-Benítez et al.^81^ assessed the effectiveness of an ANN-GA model for the evaluation and optimization of wastewaters treatment containing sulfate withbromophenol blue dye using an electro-oxidation (EO) process. In a detergent industrial WWTP, FFNN (MLP), a cascade forward neural network and SVR approaches were tested to predict the performance of the WWTP of the industry by using data collected over a period of 6 months of parameters such as of COD, BOD, TDS, TSS, and oil and grease content.^82^ The MLP has shown the best models’ performance, with a maximum correlation value for BOD (*R*^2^ = 0.99, MAE = 0.33, and RMSE = 0.49). The authors plan to implement models to optimize the performance of the WWTP in a future study. The ML model has also been applied in alternative wastewater treatment processes such as electrochemical nitrate removal. Meng et al.^83^ used the ANN model to successfully predict the electrochemical nitrate removal, presenting a maximum coefficient of determination of 0.9020. ML models have also been implemented for predicting defluorination of emergent compounds, such as per- and polyfluoroalkyl substances during their treatment and removal.^84^ This work represent the first use of ML approaches for PFAS structures, with the express goal of predicting/rationalizing C–F bond dissociation energies to support effective treatment and removal, which shows the potential of these models’ implementation in the wastewater sector.^84^

#### 3.1.2 Forecasting Influent Flow

The influent flow in a WWTP has a major impact on its operation and management. Therefore, the prediction and evaluation of wastewater inflow in WWTP by applying AI models have been the goal of several studies over the last few years. A summary of these studies is presented in Table 2.

The influent flow forecast contributes, for example, to the reduction of energy consumption by optimizing the pumps’ selection and programming.^85^ Some factors need to be considered in this type of forecast, such as the weather conditions and characteristics of the WWTP itself. Hernández-del-Olmo et al.^86^ obtained an approximately 85% accuracy in the weather soft-sensor that tells the control system of a WWTP about the present weather condition by means of the inflow characteristics with two ML algorithms: K-nearest neighbors (KNN) and random forests (RF). These weather predictions are different from the traditional ones since this soft-sensor is able to predict the weather based on the WWTP influent characteristics.^86^

The influent flow at a Wastewater Reclamation Facility in Des Moines, Iowa, was predicted using a 3-layer ANN.^87^ The model was trained using 10 months of data (influent flow, precipitation, and radar reflectivity) and tested with 5 months of data by evaluating the mean squared error (MSE) and the mean absolute error (MAE). The convergence time in the training phase was improved with the BFGS algorithm. The results showed that the forecast’s accuracy decreases as the time horizon becomes longer and that the measurement metrics increase rapidly considering a time spectrum above 30 min.^87^ In addition, the authors developed a deep neural network (DNN), more precisely, a focused time-delay neural network (FTDNN), to improve the performance of the forecast over longer periods. The DNN model depicted a better performance than ANN, with the metrics’ values having a less significant increase over longer periods.^87^

Different feature selection (FS) methods (filter, wrapper, and embedded methods) were evaluated for enhancing the prediction accuracy for TN in the WWTP influent flow. ANN, RF, and gradient boosting machine (GBM) were tested with daily time-series input parameters, such as pH, dissolved oxygen (DO), COD, BOD, TSS, volatile suspended solids (VSS), NH_4_-N, and TN concentration. Results reveal that Mutual Information, including DO, COD, BOD and NH_4_-N, had the best result rather than other FS methods. Moreover, RF and GBM revealed better performance results in comparison to ANN.^88^

To reduce the overflow in a WWTP in Drammen, Norway, Zhang et al.^89^ developed a hydraulic model to identify the spatially distributed free space and three RNNs models, Elman,^90^ NARX,^91^ and LSTM, to predict overflow in rainy situations. The input data (precipitation and flow data) of models data were normalized for the training phase in an interval between 0 and 1. For the Elman and NARX models, the authors divided the data on training, testing, and validation, by 70%, 15%, and 15% respectively.^89^ In the LSTM model, 80% of the data were used for training and 20% for testing. In the training of all models, a tuning process was carried out, based on tentative errors, from the models’ architecture to the number of hidden layers. Of the three models, LSTM performed the best to find long-term dependencies and dealing with dynamic flow changes.^89^

The RF model was used for the daily forecast of wastewater effluents in two WWTPs in Ontario, Canada.^92^ To validate the model’s performance, the authors compared the same with models using ARIMA and MLP, based on *R*^2^, NSE and the mean absolute percentage error (MAPE). In general, the RF model could forecast wastewater inputs competently, and in comparison with the ARIMA model, although in one of the stations the results were not as good as in the other, and the MAPE was smaller by about two units.^92^ Regarding the MLP model, the RF model did not capture extreme values, but the results were generally satisfactory.^92^

Szelag et al.^93^ carried out a study whose objective was to compare the application of different nonlinear methods to model the sewage flow in a WWTP in Rzeszów, Poland. The authors compared four models: RF, SVMs, KNN, and Kernel Regression. As input, the models received precipitation values, the water levels of the Wisłok river, and WWTP sewage inflow, between the period 2005 to 2008. The input variables were normalized by the min-max transformation and selected using a matrix relevant correlation. Regarding the assessment metrics of both models developed, MAE and MAPE were used. The models were tested in 12 investigations with different inputs. The authors concluded that in about 75% of the investigated cases, the SVMs method was more effective than the others and that over three inputs were always the best model. Among the 4, the Kernel Regression never managed to be the best model in any of the investigations. In both models, the authors concluded that research with the largest number of input variables showed better results at both the level of MAE and MAPE.

Recently, a multiobjective supervisory control (MOSC) strategy was conceived to optimize the wastewater treatment, under variable influent conditions in a hyperhaline wastewater treatment plant in N-city, South Korea, search optimal set points of multiple controllers.^94^ First, a fuzzy c-means (FCM) clustering algorithm distinguished specific influent conditions according to a scenario, and then for each influent condition, the DNN model estimated the WWTP performance based on the BSM2 with three WWTP local controllers: aerobic reactors, external carbon, and biogas production. Finally, the optimal set points of each controller to satisfy the desired control objectives were automatically searched by nondominated sorting genetic algorithm II (NSGA-II). The results showed that the MOSC strategy can stably contain extreme influent conditions, 8% of reduce operational costs, maintain effluent quality, and produce biogas for sustainable WWTP operation.^94^

#### 3.1.3 Anomaly and Fault Detection

Deep belief networks (DBNs) model and one-class support vector machine (OCSVM) were used with effectiveness, as a fault detection method, to monitor operating conditions of a decentralized WWTP in Golden, CO, USA.^95^ Dairi et al.^96^ developed data-driven unsupervised anomaly detection approaches, by combining the RNNs capacity to capture temporal autocorrelation features with a restricted Boltzmann machines (RBM) function to describe complex distributions. The results were validated through seven years’ influent conditions data from a coastal WWTP, Saudi Arabia, and showed the superior performance (*R*^2^ up to 0.98) of the RNN-RBM-based OCSVM approach to detect anomalies. Inoue et al.^11^ proposed an anomaly detection method for a water treatment plant based on unsupervised ML. The authors compared adapted to time series data generated by a cyber-physical system (CPS) DNN model with one-class SVM. DNN generated less false positives, while SVM detected slightly more anomalies.^11^ Overall, the DNN has a slightly better F scores than the SVM.^11^ Also, a real data set containing over 5.1 million sensor data points was used to evaluate the effectiveness of a method based on DNN (LSTM) compared to statistical and traditional ML methods (such as PCA-SVM) to model faults in the oxidation and nitrification processes.^97^ The new model performed better than the traditional methods, with a fault detection rate of around 92%.^97^ Information regarding the models’ performance in the discussed works is presented in Table 3.

#### 3.1.4 Energy Consumption Optimization

Artificial intelligence (AI) models have been used to optimize the energy consumption in WWTP. ML (NN and RF models) was used by Torregrossa et al.^98^ to develop energy cost models with high performance for WWTPs. Therefore, a database of 317 plants situated in northwest Europe was used. The model performance indicators were usually better than the ones in the literature, when the machine learning cost modeling (MLCM) algorithms were applied.^98^ This work concludes that the pollution load (COD, TP, and TN) in the inflow is the parameter with the highest impact on the energy cost of the WWTPs, and the price of energy has a minor impact on the energy consumption cost model. Also, the energy consumption of a WWTP was forecasted by LSTM, GRUs, and unidimensional CNN approaches.^99^ The results demonstrated that the pretrained univariate CNN model was the one that performed the best, presenting an approximate overall error of 630 kWh when on a multivariate setting. Oliveira et al.^99^ have successfully implemented learning processes, with the overall error reducing to 325 kWh. In addition, Bernardelli et al.^100^ described the design and field testing on a large-scale municipal WWTP of about 500,000 population equivalent of the energy way (EW) model predictive controller (MPC) based on ANFIS and a heuristic search. The model was able to predict the TN peaks (30 min in advance), allowing them to adapt the air flow and ensuring compliance with effluent discharge parameters, while saving energy. Finally, a new hybrid neural network (PCA-CNN-LSTM) model based on DNN was proposed and tested with two years’ data from a WWTP in Chongqing, China.^101^ The model was able to predict the effluent parameters and optimize energy and materials consumption, achieving reductions in total energy and materials costs around 10% to 15%.^101^ DNN were also used by Oulebsir et al.^102^ to optimize the energy consumption in WWTP using an activated sludge process. The model showed good results with a *R*^2^ varying between 90–92% in the training period and 74–82% in the testing period, and showed a gain in energy for most of the data.^102^ RF was tested as an energy consumption model, using data from 2472 WWTPs in China.^103^ The RF model had an *R*^2^ of 0.702, which was much higher than the one obtained for the multiple linear regression (0.147), therefore implying a higher accuracy.^103^ In Table 4 is a summary of the previously discussed works.

Besides directly optimizing the energy consumption, some recent works focused on using tools such as CNN, RNN (LSTM), and hybrid CNN-LSTM to predict the optimal aeration rate of dissolved oxygen that needs to be applied to the A^2^/O (anaerobic-anoxic-aerobic) process by using data of influent and effluent of COD, nitrate, and the amount of dissolved oxygen present in each biological step.^104^ These authors also established an online learning-empowered smart management of the A^2^/O process in sewage treatment processes (OL-AP). By optimizing the optimal aeration, these approaches will also minimize/optimize the energy consumption in this process.

## 4 Wastewater Treatment Modeling Using Hybrid Models

Very few examples of hybrid models (HM) applications in water and wastewater treatment are available.^105^ The first examples appeared around 2000 and were based on neural networks (Table 5). These works simulated the prediction errors of a simple MM,^106^ the nitrogen dynamics process reaction rates,^107^ and the concentration of the effluent components by comparing serial and parallel hybridization.^108^ The use of neural networks gave a very good level of interpolation but showed a poorly extrapolative capability.^108^ Thus, Lee et al.^109^ also compared the performance of different AI algorithms (Table 5) in a parallel hybridization with ASM1. They concluded that all HM tested performed better than the MM alone; however, they found high discrepancies between training and validation periods.

**Table 5:** Summary of Hybrid Model Applications Focused on WWTPs

MM model	AI algorithm	hybridization scheme	objective	model performance	ref
Activated sludge process^110^	Feedforward neural network	Parallel	Effluent SS, COD_T_, NH_4_^+^, dissolved oxygen in the mixed liquor, and VSS in digested sludge	Good accuracy of the dynamics of the activated sludge process; some of the observed deviations were explained by noisy effluent data.	(106)
Simple model describing nitrogen dynamics^111^	Neural Network	Parallel	NH_4_^+^ and NO_3_^–^	Hybrid model is very accurate and its predictions agree very well when compared with new data not used for its development; control results produced by the hybrid model were inferior to those produced by the linear model.	(107)
ASM1	Neural Network	Serial/Parallel	Predict the concentration of effluent components	Best performance of parallel hybridization in comparison with the serial hybrid model (sum of squares error of 15.25 for MM, 9.91 for NN, 12.43 for SHM and 7.58 for PHM)^b^	(108)
ASM1	Feedforward back-propagation neural network Radial basis function network Linear partial least-squares (PLS) Quadratic PLS Neural network PLS (NNPLS)	Parallel	Mixed liquor suspended solids (MLSS), COD, suspended solids (SS), and cyanide (CN)	All HM tested performed better than the MM alone. High discrepancies between training and validation periods. FBPN and RBFN presented the lowest relative sum of square error during the training period, but higher during the validation (0.018 vs 0.163 for FBNM; 0.012 vs 0.130 for RBFN)	(109)
ASM2d (GPS-X)^a^	Gaussian Process	Serial/Parallel	Effluent TN and TP	Improve the model prediction accuracy in terms of *R*^2^ and variance of the prediction error (e.g., *R*^2^ for TN of MM was 0.065 in comparison with 0.13 in serial and 0.814 in parallel hybrid model)	(105)
BSM1	Neural Ordinary Differential Equation	Parallel	Effluent NH_4_^+^	Lowest RMSE of 0.46 g/L, in comparison with 0.83 g/L of MM and 4.19 g/L of ML	(12)
ASM1	LSTM	Serial	Prediction of nitrous oxide (N_2_O) emissions	Superior prediction performance of hybrid model (MSE = 0.013) in comparison with the MM (MSE = 0.086) and ML (MSE = 0.0545)	(112)
ASM (GPS-X)^a^	RF	Serial	Effluent NH_4_^+^-N	Highest *R*^2^ of 0.95 and the lowest RMSE of 0.23 mg/L and RB of 0.2%.	(113)
ASM2d	CNN-LSTM	Serial	Effluent COD, NH_4_^+^-N	Better performance of HM (performance exceeds 7% of the baseline)	(114)
ASM (DHI WEST)^a^	Multi-Layer Perceptron (MLP) regression	Serial	Effluent COD, NH_4_^+^-N, TN, TP and energy consumption	Better anaerobic-anoxic-oxic process strategy, with energy consumption savings of about 49%	(115)

a Commercial simulation software.

b NN – neural network model; SHM – serial hybrid model; PHM – parallel hybrid model.

Serial hybridization requires that mechanistic or data driven processes are run sequentially, with the output of one being the input of other. In parallel hybridization, both models run in parallel, where, for example, the data-driven model could be trained to learn the mismatch between the mechanistic model and experimental data, reducing the residual error, or could be applied to perform the same prediction, improving the final ensemble model performance.^34^ The serial approach is mainly used to fill the gaps in input data, while the parallel structure is used to improve the model response in conditions never seen by the model. Finally, a new approach to parallel hybridization was presented by Quaghebeur et al.,^12^ by the incorporation of a neural differential equation into a mechanistic model, thus capturing the missing dynamics of the mechanistic component.

Another interesting potentiality of hybrid models is the reduction of the model calibration needs. Hvala and Kocijan^105^ reported that the prediction accuracy of the hybrid model is comparable to the tuned MM (i.e. with calibrated parameters). This could represent a huge time savings by eliminating the calibration step.

Detailed information on the use of hybrid modeling in water resource recovery facilities can be found in the recent review of Schneider et al.^34^

## 5 Gaps and Future Directions

As referenced, mechanistic models involve the development of a series of simplified mathematical formulations with the purpose of mimicking the real system. This approximation results in the loss of accuracy due to parameter and stochastic event adjustments, which propagates and aggravates the predictive performance. In this type of model, the closer to reality, the more difficult the calibration process. Indeed, most of kinetic parameters, derived from unmeasurable parameters in Monod expressions, such as inhibition constant, maximum growth rates, half-saturation constants, and substrate utilization rates, are usually determined in controlled biochemical measurements.^116^ This results in the necessity of frequent calibrations due to the complexity and variability of wastewater exposed to the microbial communities. In addition, the precise incorporation of multiple time and space scales represents a difficulty to mechanistic models.^117^ These drawbacks are overcome by data-driven models based on AI. AI models can provide universal predictions that are missing in the mechanistic models due to their oversimplified assumptions and extremely specific nature. Nevertheless, with AI and mechanistic modeling approaches different types of information can be afforded, since they rely on different types of data.^117^

In recent times, AI models have been gaining more and more impact. The last 5 years have seen an exponential increase in publications in the scientific community, considering the use of ML models within the scope of WWTPs (Figure 1). Despite this growing increase in the use of AI models in various aspects of WWTPs, there are still steps to make their applications more robust and wider. AI models can handle data sets of large capacity.^118^ In particular, in the case of DL models that aim to forecast time series, the periodicity of data capture is one of the essential parts of this process. In this aspect, all of the features that will serve as input to the AI models must have the same periodicity. Due to this factor, the data that even had considerable size, at first sight, ended up having a smaller size at the end of the entire treatment process. If this grouping is carried out for a different periodicity, some DL models, whose great asset is the ability to consider the time series present in the data, such as the LSTM, can lead to performance breakdowns. Hence, more significant temporal stability in data collection by the WWTP management entities becomes crucial to avoid the decrease of the size of the collected data set. Also, some studies conducted experiments with data sets of limited size. They achieved a performance comparison between deep learning and traditional statistical methods, such as ML algorithms. A recent study^119^ used several models, such as ordinary least square (OSL), seasonal decomposition by local regression (SDL), exponential smoothing state space (ES), and ARIMA, to predict energy consumption in WWTPs. In this study, the ARIMA model had a better MAPE performance than did the others. Other works are used to predict biochemical parameters or energy consumption in WWTPs, such as a work of Bagherzadeh et al.^88^ that did a comparative study on predicting total nitrogen in WTTPs by testing ML and DL algorithms. The results of this work show that the RF model obtained a better RMSE than other algorithms.^88^ Another work aimed to predict the dissolved nitrous oxide (N_2_O) concentration in a sequence batch reactor (SBR) by applying ML algorithms such as SVR.^120^ In the context of WWTPs, the problem of the lack of data results from the fact that some of the data are still being collected manually and require laboratory analysis, namely, data acquired by analytical control. Hence, considering the data set’s periodicity as the most frequent periodicity in its features usually leads to a decrease in its size. Despite this, many installations already have an extensive history of data and sensing at the level of analytical control of the water, which facilitates the use of DL models, which need a large set of data, namely, in the scope of forecasting time series. In addition, one of the methods that can be used regarding the size of the data set is the application of data augmentation.^121^ Through this technique, it will be possible to artificially increase, through the collected data, the size of the training data set to be used by the AI models. However, there are some limitations when using this technique. One is that the biases present in the original data set will remain in the augmented data set. Furthermore, guaranteeing quality assurance in data augmentation is expensive and time-consuming.

In time series forecasting problems, some factors can lead to a better performance of the designed models such as features in the model inputs that present a strong correlation, whether negative or positive, with the target feature intended to be predicted. In this context, not all the various studies analyzed carry out a feature selection process before applying the different conceived models. These studies could obtain better results if they only used feature models correlated with their target as input. Using features that are not strongly correlated with the target to be predicted can lead to worse performance of the model.^122^ Hence, a good feature selection leads to better performance from DL models. Another essential factor to consider in this type of problem is cross-validation. This aspect is vital as it aims to assess how the model results will be generalized to an independent data set. Through cross-validation techniques, it is possible to limit problems such as overfitting or underfitting of the conceived models.^123^ Preventing these problems is essential, so the model does not generalize to a given training data set. In the case of time series, it is also necessary to use specific cross-validation, such as time series split, so that the test data set has more recent periodicity than those used in the train. Analyzing the reviewed studies, not all consider this vital aspect when conceiving time series forecasting models (Tables 1, 2, and 4).

Regarding anomaly detection models, there is still a lack of studies in WWTPs. The WWTPs must follow limits imposed on the emission of various substances present in the wastewater, thus leading to tight control of these values. However, there may be times when this control may fail due to multiple factors, such as a failure in one of the wastewater treatment processes at these facilities. In this sense, anomaly detection models can be advantageous, alerting people who work in WWTPs to some anomalous value in some processes carried out in the facilities. We can identify a practical example of this utility in energy consumption. If any of the processes use more energy than usual, it may indicate a failure in equipment used in the process in question, causing it to consume more energy. In this case, using an anomaly detection model can help to identify this problem more quickly, leading to faster action by the WWTPs’ interlocutors. Nevertheless, to study the best anomaly detection model for different data sets, it is necessary to label them by people specialized in the area to classify a value as an anomaly or not an anomaly.

One of the aspects pointed out to data-driven models is their lack of transparency and an explanation of what happens in their process. Many companies today still have difficulty using AI models due to the lack of confidence and security in understanding the whole process. Therefore, it is essential to give interpretability to the black box that surrounds data-driven models. One of the future directions is the application of Explainable AI (XAI) to demonstrate the entire process performed within these algorithms, such as feature importance.^124^ In addition, using a Transfer Learning process is another point to consider as a direction. This process aims to use a pretrained model on a given problem, applying it to another but within the same context.^125^ For example, at the level of a WWTPs management entity, the use of a pretrained model for forecasting energy consumption in a given WWTP can be reused in a different WWTP, for a similar forecast. This way, a single trained model can be used in different WWTPs, within the same context. However, to use this process in the context of WWTPs, attention to the infrastructure will be necessary. In the case of a pretrained model for predicting energy consumption in a specific WWTP, if we use it to carry out the same prediction in a larger WWTP where overall energy consumption is higher, this type of approach will not have many effects because the model was trained in a range of smaller values due to less energy consumption.

Nevertheless, to date the majority of the literature studies are based on specific study cases, and there is a lack of benchmark calculations.^126^ Still, a few studies have looked into this issue. For example, Torregrossa et al.^98^ benchmarked the classic cost approaches with the performance of neural network and random forest to estimate the cost function in WWTP. In addition, the BSM1 platform was used to simulate a reinforcement learning-based particle swarm optimization method to optimize the control setting in the sewage process in WWTPs.^127^ The results of this approach demonstrated that the developed model could provide feasible treatment solutions while reducing the operating costs. Another benchmark calculation example was provided by Heo et al.^94^ In this study, the authors developed a hybrid machine-learning algorithm to find optimal set points of multiple controllers under varying influent conditions. They applied the BSM2 to model the WWTP and test the multiobjective supervisory control strategy.^94^

Data-driven models are built under a set of hyperparameters without any physical and biological meaning, lacking the processes’ interpretability achieved by the mechanistic models. In addition, large data sets are needed to represent the entire WWTPs’ operation, this being the only source of knowledge to the model. This fact makes model predictions difficult when the WWTPs are under environmental or process disturbances.^12^ Thus, as a future direction, we envisage the combination of both model approaches (mechanistic and data-driven), as the pros of one tend to be the cons of the other, allowing the junction of expert knowledge with data. The construction of hybrid models applicable to WWTPs could rely on an AI layer overtaking the mechanistic framework, combining data-driven models into a single loop by employing cycle-consistent adversarial networks. Thus, the mechanistic framework will facilitate the interpretation of model results, while the data-driven model can provide the individual parameter calibration and model refinement.

Furthermore, with the conception of hybrid models, it will be possible to cover some essential aspects in parallel and series approaches. Considering the hybrid models with a parallel approach, one of the objectives would be to explain the result coming from the ML models. Nowadays, ML models still have a gap in the interpretability of their results without any explanation, known as Blackbox. Using the series hybrid model design approach, the output generated by the ML models would feed the MM, with their input. Through this mechanism, the MM would explain the obtained results by the ML models, which could improve the decision-making process in WWTPs.

On the other hand, using the parallel approach for designing these models, the focus would be on minimizing the prediction error of both models. ML models perform well with a greater amount of available data. However, if we consider small data sets or disturbances in the systems, the ML models cannot perform satisfactorily, since it had small data set to be trained and may not have knowledge to predict disturbances.^34^ This would happen due to the lack of more data to learn these variations. In these cases, the MM may respond better to the variations presented in the data. By using the models in parallel, we will have a more accurate and calibrated forecast, always considering the model that obtains the best performance at the instant of time that we want to forecast.

Nowadays, some studies already use hybrid models in the field of ML, such as CNN with GRUs or CNN with LSTM, to predict energy consumption^128,129^ and Complete Ensemble Empirical Mode Decomposition with Adaptive Noise (CEEMDAN) joining algorithms XGBoost and RF, for water quality prediction.^130^ Compared with DL algorithms, this model gave better results. The principle of the hybrid model approach is to take the strengths of different models and their knowledge representations,^131,132^ as the CNN-LSTM hybrid model utilizes the ability of the CNN to extract features and LSTM to handle time series and sequence data. This combination intends to minimize the minimization of RMSE.

## 6 Conclusions

Computational modeling has shown to be a promising tool to assist in the management of WWTPs. Recent years came with a shift from the traditional mechanistic models where the process design has a special role to data-driven models, where modeling is based on machine learning approaches, without providing any knowledge about the function of the system. Nevertheless, data-driven models present better prediction capabilities than mechanistic ones, and overall, they present smaller errors.

Data collection and curation were identified as the main limitations to be overcome for a wider implementation of AI models. Prediction of influent flow and effluent characterization are the most studied applications. Nonetheless, there is room for significant developments in models for anomaly detection and energy consumption optimization, for example.

Despite the availability of mechanistic models for the different elements of water and wastewater systems, a robust integration with data-driven models is still missing to achieve an optimal balance between their prediction capabilities and the required computational power. Thus, future research should focus on the implementation of combined mechanistic and data-driven models. This approach will contribute to the economic and operational efficiency of WWTPs increasing their environmental sustainability.
